# A new RNASeq-based reference transcriptome for sugar beet and its application in transcriptome-scale analysis of vernalization and gibberellin responses

**DOI:** 10.1186/1471-2164-13-99

**Published:** 2012-03-19

**Authors:** Effie S Mutasa-Göttgens, Anagha Joshi, Helen F Holmes, Peter Hedden, Berthold Göttgens

**Affiliations:** 1 Rothamsted Research-Broom's Barn, Department of Applied Crop Science, Higham, Bury St Edmunds, Suffolk IP26 6NP, UK; 2 University of Cambridge, CIMR, Hills Road, Cambridge CB2 0XY, UK; 3 Rothamsted Research, Department of Plant Science, Harpenden, Herts AL5 2JQ, UK; 4 School of Life Sciences, University of Hertfordshire, Hatfield, Herts AL10 9AB, UK

## Abstract

**Background:**

Sugar beet (*Beta vulgaris *sp. *vulgaris*) crops account for about 30% of world sugar. Sugar yield is compromised by reproductive growth hence crops must remain vegetative until harvest. Prolonged exposure to cold temperature (vernalization) in the range 6°C to 12°C induces reproductive growth, leading to bolting (rapid elongation of the main stem) and flowering. Spring cultivation of crops in cool temperate climates makes them vulnerable to vernalization and hence bolting, which is initiated in the apical shoot meristem in processes involving interaction between gibberellin (GA) hormones and vernalization. The underlying mechanisms are unknown and genome scale next generation sequencing approaches now offer comprehensive strategies to investigate them; enabling the identification of novel targets for bolting control in sugar beet crops. In this study, we demonstrate the application of an mRNA-Seq based strategy for this purpose.

**Results:**

There is no sugar beet reference genome, or public expression array platforms. We therefore used RNA-Seq to generate the first reference transcriptome. We next performed digital gene expression profiling using shoot apex mRNA from two sugar beet cultivars with and without applied GA, and also a vernalized cultivar with and without applied GA. Subsequent bioinformatics analyses identified transcriptional changes associated with genotypic difference and experimental treatments. Analysis of expression profiles in response to vernalization and GA treatment suggested previously unsuspected roles for a RAV1-like AP2/B3 domain protein in vernalization and efflux transporters in the GA response.

**Conclusions:**

Next generation RNA-Seq enabled the generation of the first reference transcriptome for sugar beet and the study of global transcriptional responses in the shoot apex to vernalization and GA treatment, without the need for a reference genome or established array platforms. Comprehensive bioinformatic analysis identified transcriptional programmes associated with different sugar beet genotypes as well as biological treatments; thus providing important new opportunities for basic scientists and sugar beet breeders. Transcriptome-scale identification of agronomically important traits as used in this study should be widely applicable to all crop plants where genomic resources are limiting.

## Background

Sugar beet crops account for about 30% of world sugar production and are important in Europe, North America, and increasingly in Asia, South America and North Africa. In temperate climates, sugar beet is grown as a spring crop, whereas in warmer climates, such as in the Californian Imperial Valley, it is grown as a winter crop having been sown in the autumn. There is increasing interest in developing winter crop varieties for cultivation in the cooler temperate regions. It is estimated that extending the growing season by autumn sowing in these regions could result in at least a 26% yield advantage [1], offering opportunities for additional applications for sugar beet as a sustainable feedstock for bio-fermentation processes. A key breeding target for both autumn and spring sown crops is the suppression of cold temperature induced stem elongation (bolting) and flowering (reproductive growth) during the growing season. This is because, in sugar beet crops, prolonged exposure to cold temperatures in the range 6°C to 12°C [2], a process known as vernalization, is obligatory for the induction of reproductive growth, which requires that the plants must first bolt and then flower. An incidence of one premature bolting plant per square metre in the field can cause a 12% loss in root sugar yield [3]. Improved knowledge of the vernalization mechanism is widely regarded as an important prerequisite for the identification of new breeding targets. Currently, the key breeding strategy is to select against the early bolting gene *B *[4], thereby maintaining the biennial habit so that crops remain vegetative as long as temperatures do not become vernalizing during the growing season.

Attempts to find alternative breeding targets are largely reliant on reverse genetics approaches, whereby putative sugar beet flowering genes are identified based on homology with counterparts of the *Arabidopsis *model. This has uncovered several factors [5,6], including some which have been shown to affect vernalization responses [7]. The role of gibberellins (GAs) has also been examined and it has been demonstrated that there is an interaction between vernalization and GA-dependent bolting responses although the underlying mechanisms are not known [8]. A picture is beginning to emerge for gene regulatory networks in sugar beet, in which genes homologous by sequence and protein function to their *Arabidopsis *counterparts are not necessarily conserved with respect to their developmental roles [5,7]. It is therefore important to study vernalization directly in sugar beet in order to gain new mechanistic insight. Comprehensive transcriptome-scale analysis of sugar beet is complicated by the fact that there is no reference genome and also no commercial array platforms for expression profiling. The only public resource for sugar beet gene sequences is the sugar beet plant gene index database of EST collections at http://compbio.dfci.harvard.edu/cgi-bin/tgi/gimain.pl?gudb=beet. Recent breakthroughs in next generation sequencing technology and data analysis suggest that it is now possible to generate a reference transcriptome in the absence of a reference genome [9], and then to use this reference transcriptome to perform comparative expression profiling by methods such as digital gene expression profiling. This novel technology therefore offers exciting new opportunities to crop geneticists who hitherto had to rely on a handful of model plant species for transcriptome-scale studies. The physiological characteristics of these species are often very different from the crop under investigation, thus, making them less than ideal model systems.

Here, we report a transcriptome-scale analysis of the transcriptional programs in sugar beet plants subjected to either vernalization, GA treatment or a combination of both. The analysis was restricted to the shoot apex, which includes apical meristematic tissues within which the developmental transitions leading to flowering are known to occur [10]. We therefore isolated shoot apices by micro dissection from appropriately treated plants and subsequently extracted total RNA for mRNA sequencing. We selected the next generation HiSeq2000 technology platform, with the intention of (i) constructing an assembly of our shoot apex transcriptome; (ii) conducting a digital expression profile analysis of transcripts in each sample; (iii) mapping the expressed transcripts back to our reference transcriptome and (iv) gaining insight into the potential key candidates underlying vernalization and GA-dependent responses in sugar beet. In addition to gaining knowledge of new sugar beet genes, we also expected to conduct an assessment of our strategy as a method for transcriptome-scale analyses of agronomically important traits in sugar beet, as an example of the potential for application in other crop plants.

## Results

### Sample generation and sampling strategy for analysis of vernalization-induced and GA-dependent gene expression in shoot apices

Sugar beet plants are out-breeding, with a tendency to suppress self-compatibility and therefore are naturally highly variable at the population level. This makes it difficult to interpret genetic data unless the experimental populations are fixed to some extent. The level of genetic variability can be reduced by selecting lines that are generated through single seed decent. To achieve this, we first selected C600 lines of the genotype *bb*, lacking the early bolting gene *B *and therefore unable to bolt without prior vernalization. These lines are considered to carry the late bolting gene *Lb*-*lb *[11] which is known to be linked to the monogerm character [12]. Siblings from one of these C600 bb lines were grown to maturity, vernalized for 18 wks and scored for bolting and flowering time. Sibling plants which bolted and flowered within 1-2 days of each other and designated C600 MB1-7; C600 MB1-13 and C600 MB1-35 were inter-crossed to provide bulk seed for our experiment. This bulked seed lot, designated C600 MB1 SibA, was then used to raise plant material for the experiment described here. In our hands, the generation time of biennial plants can be reduced to 1 year by artificial vernalization, in a controlled environment chamber, without compromising seed quality and quantity. Thus it took just over 2.5 yrs (including seed maturation/drying) to generate material suitable for our experiment. A second *bb *genotype, Roberta, a proprietary commercial cultivar was included in our experiment. We could then evaluate and quantify differences and/or similarities between our experimental line and commercial varieties, which are normally generated as hybrids of 3 different genetic backgrounds, combined in 2-way crosses between F1 cytoplasmic male sterile plants and a pollinator line [13].

**Figure 1:**
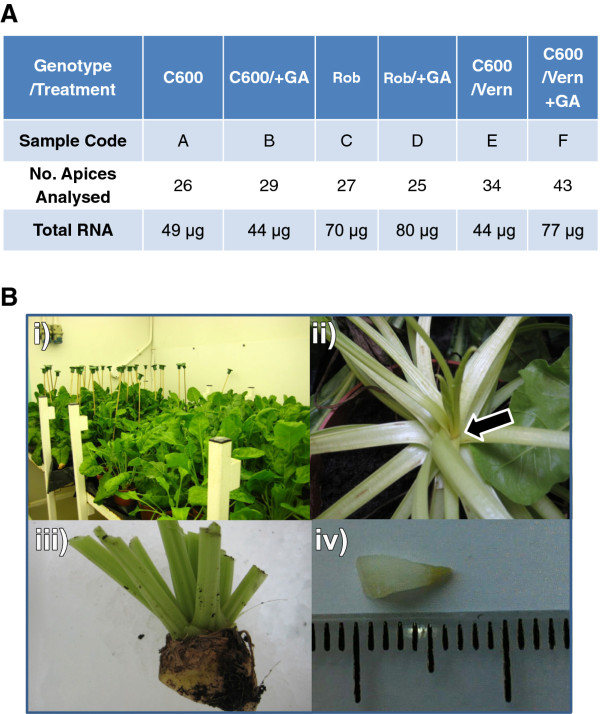
**Experiment overview and sampled tissues**. A) Different sugar beet genotypes C600 and Roberta (Rob) were kept in short days (8 h photoperiod) and treated with GA_4_, added by pipette directly to the shoot apex without having been vernalized (/+GA) or after having been vernalized at 6°C for 18 weeks (/vern + GA). Shoot apices were pooled from individual plants prior to RNA isolation to allow sufficient material for robust RNA purification. The total number of apices analysed/treatment is indicated, as are the final yield of total RNA. The RNA-Seq method used required a minimum of 30 μg of total RNA/sample. B) The picture shows a typical example of the developmental stage and condition of plants when sampled - i) view of plants in the growth chamber with the GA-treated plants in the background marked with wooden canes; ii) a close up of the shoot tip, arrow; iii) example of plant apices as harvested; ii) typical example of the shoot apex after dissection, next to a ruler with 1 mm divisions.

It is generally accepted that vernalization alters meristem competence to flower [14] and, in sugar beet evidence exists to suggest that vernalization signals are perceived in the leaves [15]. Further, our previous studies (unpublished) have indicated that vernalization-dependent GA-induced developmental processes leading to reproductive growth appear to be localized to the apical shoot meristematic tissues. The role of GA in floral regulatory networks is well established [16] and has been demonstrated for bolting and flowering in sugar beet [8,17]. To perform transcriptome-scale analysis of associated changes in gene expression, we harvested between 30-50 plants per treatment and micro-dissected apical tissues (Figure 1B), under the stereo microscope. A total of 184 apices were used in this experiment, distributed amongst the treatments as indicated (Figure 1A). Dissections were carried out ensuring that as much of the vascular and leaf tissues as possible were removed, whilst taking care to retain the meristem (Figure 1B iv). Following total RNA extraction, in-house quality assurance was carried out by first ensuring that there was no genomic DNA contamination. This was achieved by conducting a no-RT endpoint PCR reaction targeted at the housekeeping *BvEF1a *gene, using primers: L1: GATTCCCACCAAGCCTATGG and R1: GATGACACCAACAGCGACAG, optimised at 150 nM and 60°C. Next, the integrity of the RNA samples was confirmed on a standard denaturing formaldehyde RNA gel and by *BvEF1a *RT-PCR. Minimum 30 μg aliquots of total RNA for each treatment were then sent for custom sequencing prior to which the samples were quality controlled further by the service provider.

### A sugar beet reference transcriptome generated by mRNA-sequencing

**Figure 2:**
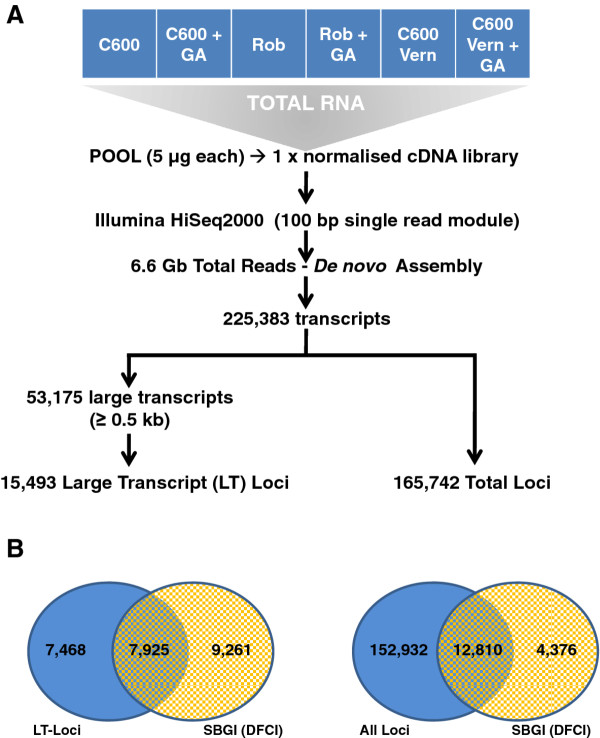
**Overview of the reference transcriptome sequencing, and result of the Velvet/Oases assembly**. A) RNA for the reference was pooled from all of the test samples. The Illumina HiSeq2000 platform was used to generate data for *de novo *assembly including large transcripts (LT) equivalent to 15,493 potential protein coding sequences. B) The accuracy and integrity of the assembly was assessed by BLAST comparison (100 bp overlap and ≥ 98% sequence identity) with the publicly available collection of sugar beet ESTs at the Sugar Beet Gene Index (SBGI), hosted at the Dana Farber Cancer Institute (DFCI).

Digital gene expression (DGE) profiling using next generation sequencing depends on a reference transcriptome, which was not available for sugar beet prior to this study. Pooled total RNA from all six samples was therefore used to generate a normalised cDNA library which was then sequenced using the Illumina HiSeq2000 platform (100 bp single read module - see Figure 2A). The application of short reads is now significantly improved by the increase in read length to 100 bp, such that it now provides high throughput and good value for money and is therefore commonly used for *de novo *transcriptome assemblies in non-model species [18-20]. Here, this generated a total of 6.6 Gb of sugar beet transcript sequences. *De novo *assembly using the software tool Velvet/Oases [21] and http://www.ebi.ac.uk/~zerbino/oases/, yielded a total of 225,385 unique transcripts which corresponded to 165,742 unique loci. The assembly software tools were chosen because of their previous application for the assembly of similar RNA -seq data sets [19]. In this first pass assembly, the N50 value for all loci was 1185 bp and for large transcript loci, 1573. A BLAST search of the assembly against itself revealed that there was no redundancy although we found that 250 of the 17,186 unique entries (as of 17 March 2011 update) in the public sugar beet EST database (EST-DB) hosted at http://compbio.dfci.harvard.edu/cgi-bin/tgi/gimain.pl?gudb=beet mapped to more than 1 of the large transcript loci. To verify our assembly further, we performed a second pass assembly using Minimus [22], which gave an assembly with an N50 value of 1678 bp. In contrast to the Velvet/Oases assembly, a significant proportion of these "doubly assembled" loci mapped to multiple Arabidopsis peptide sequences, thus suggesting that the Minimus assembly comes at the potential cost of over-merging, a feature that has been previously reported for peas [23]. We therefore elected to use the Velvet/Oases assembly in order to retain maximum information for subsequent analysis. The Minimus assembly output is however freely available (Additional file 1). Since there is as yet no consensus on the single best algorithm for sequence assembly [24,25], we have made our raw data (Accession ID ERP000947 in the European Sequencing Archive at EBI) freely available to the scientific community for reassessment as new tools come on line. In the meantime, our Velvet/Oases assembly is also available in Additional file 2.

In order to focus subsequent analysis on those transcripts/loci most likely to correspond to protein coding genes, we also determined the number of large transcripts (> 0.5 kb) which, in our *de novo *assembly would have required at least 6 independent 100 bp reads. We identified a total of 53,175 large transcripts, in the size range 0.5 to 8.729 kb, corresponding to 15,493 loci and hence putative protein coding sequences. To further substantiate our hypothesis that these were coding sequences, we explored the overlap between our *de novo *assembled loci and "large transcript loci" with the sugar beet EST-DB (as above), largely identified by conventional Sanger sequencing of EST collections from different sugar beet tissues. BLAST sequence similarity searches [26,27] set to a stringency of 100 bp overlap and 98% sequence identity of all 17,186 sugar beet EST-DB entries against all our 15,493 large transcript loci identified 7,925 loci common to both (Figure 2B), that is 46% of all the unigene ESTs currently in the public sugar beet EST-DB. This overlap rose to 12,810 unigene EST sequences, equivalent to 75% of the sugar beet EST-DB entries, following a comprehensive BLAST analysis with all our 165,742 shoot apex transcriptome loci.

Taken together, this analysis demonstrates a significant overlap of our new transcriptome with the existing sugar beet EST database. Moreover, the 7,568 large transcript loci with no matches in the current sugar beet EST-DB (Figure 2B) represent potential candidates of previously unknown (novel) sugar beet genes although, the possibility that some of these sequences may be the result of mis-assembly cannot be overlooked. Finally, our finding that 4,376 unigene ESTs in the existing public sugar beet database have no corresponding matches in our transcriptome is not unexpected since the public EST database is assembled from a wider range of tissue sources and experimental conditions, in contrast to the single source of shoot apices used in the current study. We elected not to perform a full-scale amalgamation of our transcriptome with the public EST database and to instead provide our database as a uniquely defined resource for vernalized and GA-treated shoot apices. In this way, it is more readily available to both breeders and academics with a specific interest in gene discoveries associated with the induction of reproductive growth in sugar beet. To facilitate such exploitation of our dataset further, we have mapped all loci to the Arabidopsis proteome by BLASTX alignment (cut off 1 × 10^-10^) with the 27,416 peptide sequences in the TAIR database (version TAIR10), and have made these data freely available (see Additional file 3).

### Digital gene expression profiling enables transcriptome-scale analysis of shoot meristem transcriptional programmes in sugar beet

**Figure 3:**
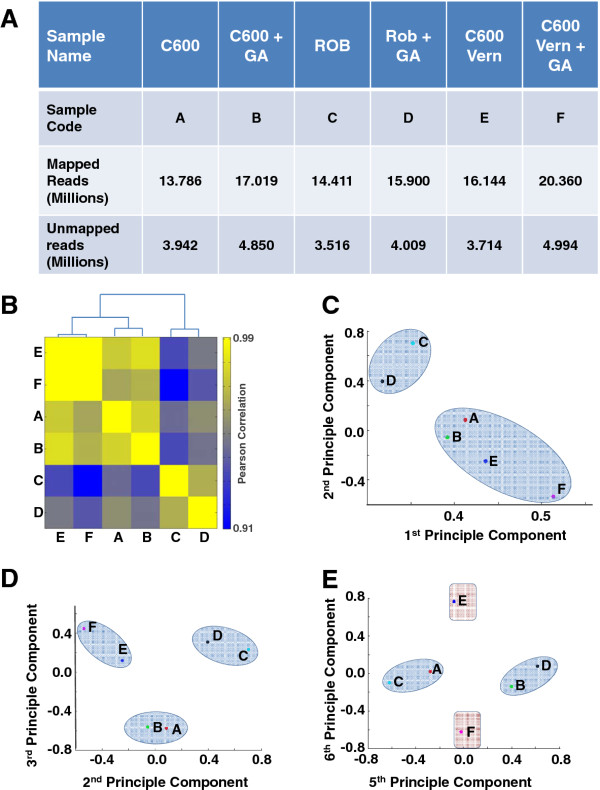
**Global analysis of digital gene expression profiles**. A) The total number of reads that were mapped back to the reference transcriptome, together with unmapped reads, for each genotype and the sample codes designated to each treatment. B) Hierarchical clustering of digital gene expression profiles for samples shown in A) reveals a major influence of genotype on global gene expression levels. Pearson correlation coefficients were calculated for all pairwise comparisons, and displayed as a heatmap following unsupervised clustering. C) Principle component analysis of digital gene expression matrix (see methods). Principal components 1 and 2 separate samples based on genotype. D) Principle component analysis displaying components 2 and 3 which separate the vernalized and non-vernalized C600 samples. E) Principle components 5 and 6 separate samples based on GA treatment (A/C *vs*. B/D and E *vs*. F).

Having generated a sugar beet shoot meristem reference transcriptome, our next goal was to perform quantitative comparisons of the transcriptional programmes in shoot apices with respect to vernalization and/or GA treatment. We also aimed to investigate the potential impact of genotypic differences. To achieve this, un-normalised cDNA libraries were generated from subsamples of the same RNA as originally used to generate the reference and extracted from apices of the genotypes C600 variously treated with vernalization and GA, and, non-vernalized Roberta, variously treated with GA. A total of six independent libraries consisting of C600/untreated (sample A); C600/GA treated (sample B); Roberta/untreated (sample C); Roberta/GA treated (sample D); C600/vernalized (sample E) and C600/vernalized and GA treated (sample F), as shown in Figure 1A, were sequenced using the Illumina HiSeq2000 50 bp single read module. For each library/sample, the total number of counts for each sequencing read were determined and the reads were mapped back to our newly established reference transcriptome using Bowtie software [28]. Mapping programs continue to evolve [29,30] hence, we are mindful of the fact that slightly different results may be obtained with alternative mappers. Nevertheless, in this study, between 13.786 million and 20.360 million uniquely mappable reads, representing between 71% - 77% of total reads, were obtained from the six libraries (Figure 3A) thus, providing good coverage for the differential expression profiling.

We next generated a matrix containing the tag counts for each locus in each of the six samples and to reduce bias due to the slight variation in sequencing depths between samples (Figure 3A), for each sample, the tag count as a ratio of the total number of mappable reads was multiplied by 10 million, thus normalizing the values to tag counts per 10 million mappable reads. To perform the subsequent global comparative analysis, only the loci with a tag count ≥ 10 in at least 1 sample were retained. This resulted in a 7-column matrix (Locus ID plus tag counts for six samples) with 23,460 rows being equivalent to the total number of loci with a tag count ≥ 10 in at least 1 sample (Additional file 4). Analysis of this matrix by hierarchical clustering based on Pearson correlation coefficients showed high correlation (0.91 - 0.99) between all 6 samples, consistent with their shared tissue origin (Figure 3B). Within this, the biggest separation was observed for the two Roberta samples, suggesting that genotype had a greater overall impact on the global transcriptome than either GA or vernalization (Figure 3B). To further explore the impact of genotype, vernalization and GA treatment on shoot apex transcriptional programmes, we next analysed our 6 digital gene expression profiles by principal component analysis (PCA). In agreement with hierarchical clustering, a PCA plot based on principal components 1 and 2 resulted in a separation based on genotype (Figure 3C). However, a PCA plot based on principal components 2 and 3 showed a clear separation on the 3rd component (Y-axis) between the non-vernalized and vernalized C600 samples A, B and E, F respectively (Figure 3D). Moreover, principal component 5 further separated the non-vernalized samples (irrespective of genotype), according to GA treatment (samples A, C and B, D) while principal component 6 clearly separated the vernalized C600 samples (E, F) according to GA treatment (Figure 3E). Taken together therefore, transcriptome-scale analysis of the digital gene expression profiles suggested that transcriptional responses to treatment can be revealed from the data generated here. Our data also revealed a major impact of genotype on shoot apex transcriptional programmes. This is important because vernalization and GA treatment induce reproductive growth [8]. Currently, it is generally accepted that response to these inductive treatments is affected by genotype although it is not clear how. The data reported in this study provide a platform for future experimentation to reveal the molecular basis of genetic components that influence bolting and flowering.

### Characterisation of genotype-driven expression differences in sugar beet shoot apex transcriptomes

Having identified genotype as the main factor determining transcriptional variation in our 6 datasets, we next set out to determine a gene set that showed high confidence expression differences between C600 and Roberta genotypes. To achieve this, we took advantage of the fact that our analysis included 4 × C600 and 2 × Roberta samples, which allowed us to determine statistical confidence scores for any potential expression differences driven by the 2 genotypes. In other words, we were able to exclude the additional variability due to treatment, thus enabling the assignment of higher statistical significance to those genotype-driven expression differences that are not also affected by GA treatment or vernalization.

**Figure 4:**
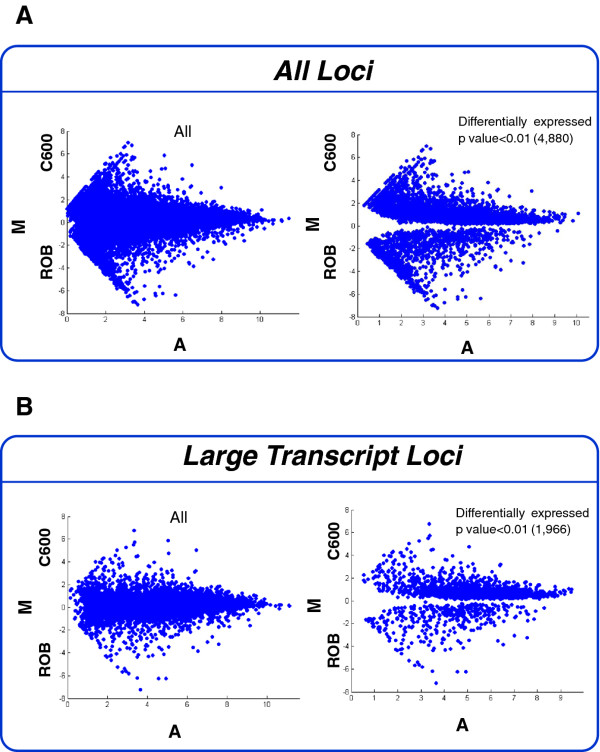
**MA plots to show transcriptome-scale difference in genotype-dependent gene expression for loci with a normalized cut off of ≥ 10 tags in at least 1 test sample**. A) Expression of all 23,460 loci in C600 versus Roberta genotype, of which 4,880 were differentially expressed as indicated. Expression values (normalised tag counts) were plotted on a log scale, so that the difference in expression is M = log_2_R - log_2_G and the average expression is A = 1/2 × (log_2_R + log_2_G); where R = C600 and G = Rob. B) Expression of all 13,125 large loci (> 500 bp with a tag count of > 10 in at least 1 sample) in C600 versus Roberta of which 1966 were differentially expressed as indicated.

As shown in Figure 4A, most loci fall on or close to the horizontal intersecting the Y-axis at zero when plotting the average expression scores in the C600 versus Roberta genotype. However, 4,880 loci showed differential expression at a p-value < 0.01 which corresponds to 21% of the 23,460 loci analysed. When the same analysis was repeated for the large transcript loci, 1,966 were differentially expressed at p-value < 0.01, corresponding to 15% of the 13,107 large transcript loci with an expression tag count ≥ 10 in at least 1 sample (Figure 4B). A bias towards C600 was observed in the differentially expressed loci which we suspect may have been a reflection of the higher number of C600 samples in our analysis. Collectively, the analyses performed here clearly demonstrate that the transcriptome datasets generated for the current study enable the global identification of genotype-specific expression differences. All differentially expressed gene loci presented in Figure 4 are listed in Additional file 5.

### Transcriptome-scale characterisation of the transcriptional response to GA and vernalization

**Figure 5:**
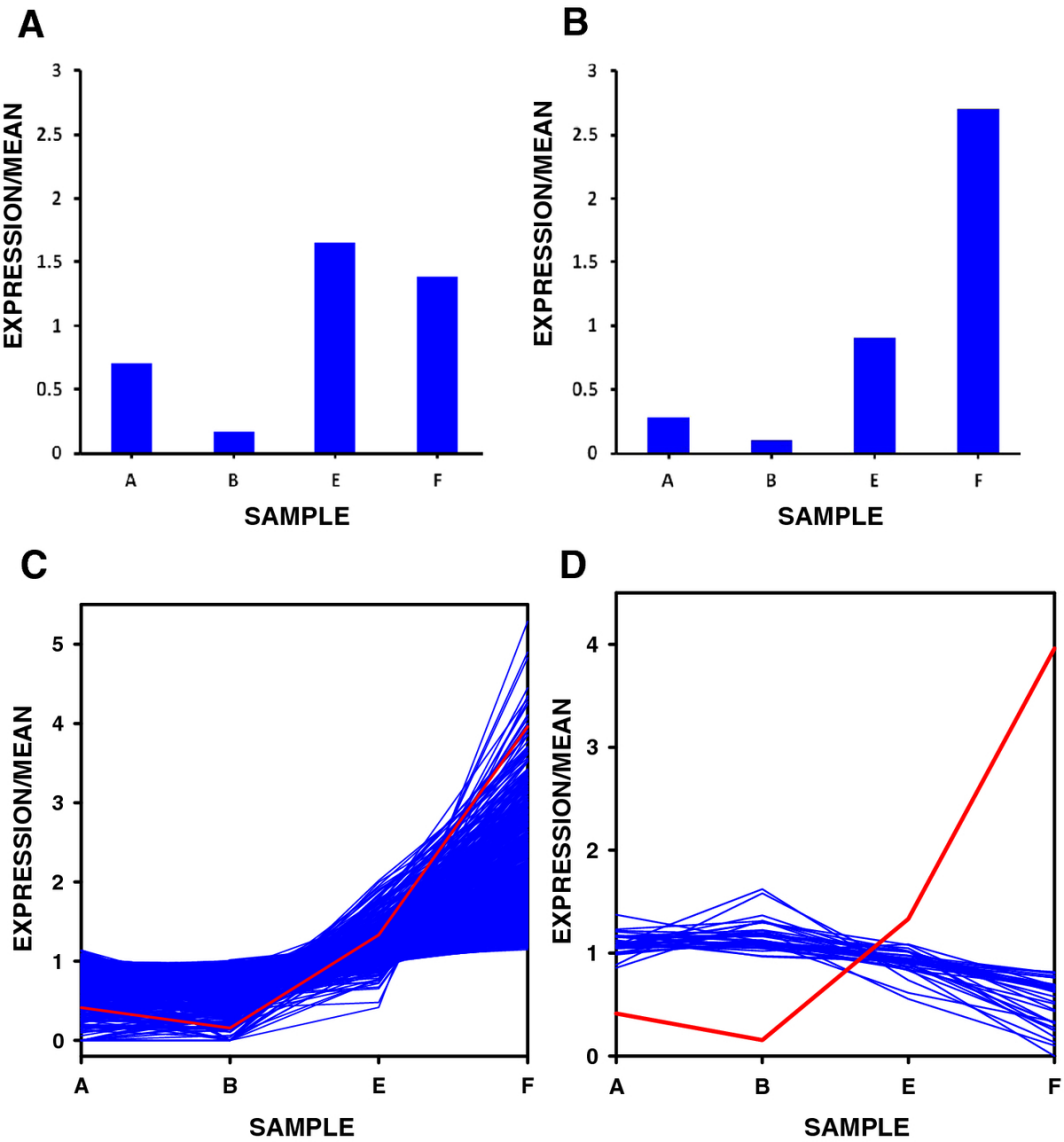
**Expression profiles of functionally annotated and novel genes in the sugar beet shoot apex**. A) Up-regulation of *BvGA20ox1 *(Locus 24372; DQ864510.1) in vernalized C600 samples. The normalised (per 10 million reads) DGE profile tag counts were 8 (sample A), 2 (sample B), 19 (sample E) and 16 (sample F). B) Up-regulation of *BvRAVL1-like *(locus 29609) in vernalized C600 samples. The normalised (per 10 million reads) DGE profile tag counts were 147 (sample A), 55 (sample B), 474 (sample E) and 1410 (sample F). C) Plot to show all loci (blue lines) that are positively correlated (p > 0.95) with *BvRAV1-like *(red line) and up-regulated by vernalization in C600 samples. D) Plot to show all loci (blue lines) that are negatively correlated (p > 0.95) with *BvRAV1-like *(red line) and down-regulated by vernalization. A = Sample C600; B = Sample C600/+GA; E = Sample C600/vern; F = Sample C600/vern + GA. Expression was relative to the mean of all samples.

Having demonstrated that the transcriptome data generated for the current study allowed for the identification of genotype-specific differences in transcriptional programmes, we next explored whether the comparison of untreated shoot apices with GA-treated and vernalized samples would allow us to define responses to these two treatments at the transcriptome-scale. Comparisons were conducted only in the C600 genotype because it had the complete set of treatments. To this end, we performed BLAST searches against our reference transcriptome using cDNA sequences from putative sugar beet candidate regulators of bolting and flowering originally cloned in-house and/or identified by *in silico *BLAST searches of the sugar beet EST sequences in public databases (Sugar Beet Gene Index at http://compbio.dfci.harvard.edu/cgi-bin/tgi/gimain.pl?gudb=beet and GenBank) using known sequences from the Arabidopsis model. Of particular interest were GA metabolism genes, and apical shoot meristem identity genes expected respectively to participate in stem elongation (bolting) and to indicate the transition to flowering [10,16]. The expression profiles of matching gene loci with at least 100 bp overlap and ≥ 98% sequence identity were selected and then interrogated against our DGE dataset. This revealed that a greater change in expression profiles occurred in response to vernalization than to applied GA. Amongst the GA metabolic genes, we found that *BvGA20ox1 *(GenBank: DQ864510.1), which mapped to our Locus 24372, was up-regulated by vernalization, with a ~2-fold increase in expression in vernalized C600 apices (Figure 5A). Amongst the meristem identity genes, our data revealed the up-regulation of a MADS domain protein, which mapped to Locus 6819 with strong homology to FRUITFUL-like sequences (e.g. the sugar beet GeneBank: BQ584677 - BLASTN p value = 4.6 × 10^-107 ^and spinach GenBank: ACE75945.2 - BLASTX p value = 1 × 10^-102^). Locus 6819 maps to the Arabidopsis *APETALA 1 *gene (TAIR: AT1G69120.1, BLASTX p value = 2 × 10^-70^) although it does not show significant similarity to the sugar beet cDNA recently deposited at GenBank, labelled AP1 (GenBank: HQ454504.1). MADs domain transcription factors like APETALA1 are, in dicotyledonous plants, generally associated with floral organ development and therefore downstream of the floral transition [31].

To interrogate vernalization responses further, we used our DGE profile matrix to rank transcripts according to expression levels in vernalized apices. We selected only those transcripts that were expressed in both non-vernalized and vernalized samples and discovered that a *RAV1 *(Related to ABA-insensitive 3/viviparous1) homologue, mapping to Locus 29609, now designated *BvRAV1-like *was, after Locus 6819, the most highly up-regulated transcript by vernalization. BLASTX analysis of this *BvRAV1-like *Locus 29609 showed that it contained 49% sequence identity to the Arabidopsis *RAV1 *(GeneBank: Gene ID 837886) over 256 amino acids and 66% similarity, with an E-value of 4 × 10^-68 ^(Additional file 6: Figure S1A). This was further substantiated by *in silico *conserved protein domain analysis, which revealed that this sugar beet homologue contained the characteristic AP2/B3 domains respectively at N- and C-terminal positions (Additional file 6 Figure S1B) as found in RAV1 [32]. The AP2/B3 domain proteins are known to bind DNA in a sequence specific manner [32] and although the function of RAV1 is not known, it is generally recognised as having a repressive effect on plant growth and development, including flowering [33]. In Arabidopsis, *RAV1 *transcripts are known to be immediately (within 1 hr) up-regulated on exposure to cold temperature [34], and are thought to play an important role in the cold stress response pathway, most likely as a component of the CBF regulon [35]. To the best of our knowledge, RAV1 has not until now been associated with vernalization responses. Here, we show for the first time that *BvRAV1-like *was stably up-regulated (2.5-fold) in vernalized apices (Figure 5B Sample E *vs*. Sample A), a response that is enhanced (~3-fold) in response to applied GA (Figure 5B Sample F *vs*. Sample E). In the absence of vernalization, our data indicate that *BvRAV1*-like was down-regulated by GA (Figure 5B Sample B *vs*. Sample A).

### *BvRAV1-like *expression is negatively correlated with genes associated with cold responses and reproductive growth

We next used our DGE matrix to identify all gene loci whose expression profiles across our C600 samples either positively or negatively correlated with *BvRAV1*-*like *(see Figure 5C and 5D, respectively). For a p-value > 0.95, 732 genes showed positive correlation, and 34 genes showed negative correlation (all gene sets are listed in Additional file 7), thus demonstrating that this approach can readily identify gene sets with highly correlated expression profiles. Given that *BvRAV1*-like encodes a putative repressive transcription factor, with a negative regulatory role on flowering [33], we suspected that amongst the correlated genes may be candidates with recognised functions in reproductive growth, including some which may be direct targets of BvRAV1. To test this, we analysed the smaller negatively correlated 34 gene set by first mapping each of the 34 transcripts to the Arabidopsis proteome, to identify homologous gene loci and selected those with E-value scores of 1 × 10 ^-10 ^or less. A total of 20 transcripts satisfied this criterion as indicated in Table 1 and were further analysed by proxy, using the best matched Arabidopsis gene locus IDs to conduct Gene Ontology (GO), Plant Ontology (PO) and network analysis using AraNet [36], to enable inference of putative gene function and regulatory networks. A total of 18 out of 21 (including *BvRAV1*) of the Arabidopsis gene homologues were present in the AraNet database and as indicated in Table 2 the associated GO terms, by collective analysis of the gene set, were enriched for plant terms including some that we considered to be consistent with reproductive growth (e.g. as for AT4G2600, LOB, RAV1) and plant development (e.g. as for AT5G14450-lipid metabolism; AT1G68830-starch metabolism; LTP4; AT1G16070-transcriptional regulation; AT3G58690-protein modification) as well as hormone responses (MYB30; AT2G45640-abscisic acid response). This was supported further by GO analysis of the 20 query genes negatively correlated with the *BvRAV1-like *homologue, in combination with the top 200 associated new candidate genes revealed by AraNet. In this case, the 39 PO enriched terms included at least 13 developmental processes that could be directly associated with reproductive growth (see Table 3). Next, we conducted more specific GO analysis of individual query loci, based on inferences from direct assays, mutant phenotype, genetic interaction, physical interaction, expression patterns and traceable author statement. This enabled the assignment of putative gene functions based on network neighbours. Consequently, (as indicated in Additional file 8) we found that loci such as AT3G58690, AT4G25720, for which there was previously no functional information (from the gene set analysis - Table 2), may also be involved in processes associated with reproductive growth including regulation of meristem organisation, primary shoot apical meristem specification and flower development; while AT1G56580 is associated with GA biosynthesis, signalling and cell growth. Full details of enriched GO terms by inference from network neighbours for each of the 18 genes are given in Additional file 8 which also shows the 6 (out of 3,063) generally enriched (p < 0.05) GO terms (out of 3,063) and the 57 (out of 5,048) enriched (p < 0.05) InterPro Domains for the 20 query genes and their associated new candidates from AraNet.

**Table 1:** BvRAV1-like transcript locus together with associated negatively regulated transcript locus IDs together with their best matched homologous Arabidopsis gene loci and their annotations

Transcript locus ID	Arabidopsis locus ID	BLASTX p-values	Arabidopsis locus annotations
29609	AT1G13260.1	4 × 10^-60^	RAV1, EDF4 | related to ABI3/VP1 1 | chr1:4542386-4543420 FORWARD LENGTH = 344
1313	AT5G63660.1	1 × 10^-25^	LCR74, PDF2.5 | Scorpion toxin-like knottin superfamily protein | chr5:25485692-25486062 FORWARD LENGTH = 73
5840	AT2G45640.1	5 × 10^-45^	SAP18, ATSAP18 | SIN3 associated polypeptide P18 | chr2:18799881-18801323 REVERSE LENGTH = 152
2345	AT3G42170.1	4 × 10^-60^	BED zinc finger;hAT family dimerisation domain | chr3:14321838-14323928 FORWARD LENGTH = 696
14853	AT3G58690.1	8 × 10^-145^	Protein kinase superfamily protein | chr3:21709369-21711246 FORWARD LENGTH = 400
14886	AT1G80480.1	1 × 10^-54^	PTAC17 | plastid transcriptionally active 17 | chr1:30258272-30260570 REVERSE LENGTH = 444
25243	AT5G63090.2	6 × 10^-59^	LOB | Lateral organ boundaries (LOB) domain family protein | chr5:25308723-25309283 REVERSE LENGTH = 186
2141	AT5G14450.1	2 × 10^-89^	GDSL-like Lipase/Acylhydrolase superfamily protein | chr5:4658488-4660034 FORWARD LENGTH = 389
18419	AT5G19300.1	6 × 10^-119^	CONTAINS InterPro DOMAIN/s: Nucleic acid-binding, OB-fold-like (InterPro:IPR016027), Protein of unknown function DUF171 (InterPro:IPR003750); Has 3649 Blast hits to 1964 proteins in 291 species: Archae - 113; Bacteria - 121; Metazoa - 1082; Fungi - 399; Plants - 227; Viruses - 4; Other Eukaryotes - 1703 (source: NCBI BLink). | chr5:6495593-6497987 FORWARD LENGTH = 398
12175	AT1G69830.1	5 × 10^-172^	ATAMY3, AMY3 | alpha-amylase-like 3 | chr1:26288518-26293003 REVERSE LENGTH = 887
98654	AT1G16070.2	2 × 10^-106^	AtTLP8, TLP8 | tubby like protein 8 | chr1:5511899-5513779 REVERSE LENGTH = 398
32761	AT1G56580.1	8 × 10^-41^	SVB | Protein of unknown function, DUF538 | chr1:21198402-21198902 REVERSE LENGTH = 166
27453	AT5G61800.1	3 × 10^-67^	Pentatricopeptide repeat (PPR) superfamily protein | chr5:24830054-24831553 REVERSE LENGTH = 499
6330	AT4G25720.1	1 × 10^-19^	ATQC, QC, QCT | glutaminyl cyclase | chr4:13099929-13102470 REVERSE LENGTH = 320
19953	AT1G64570.1	5 × 10^-10^	DUO3 | Homeodomain-like superfamily protein | chr1:23978868-23983925 FORWARD LENGTH = 1239
12524	AT5G59310.1	2 × 10^-27^	LTP4 | lipid transfer protein 4 | chr5:23925296-23925772 REVERSE LENGTH = 112
17933	AT4G26000.1	9 × 10^-104^	PEP | RNA-binding KH domain-containing protein | chr4:13197280-13199539 FORWARD LENGTH = 495
269	AT5G23850.1	0	Arabidopsis thaliana protein of unknown function (DUF821) | chr5:8038126-8040741 FORWARD LENGTH = 542
24056	AT2G26680.1	3 × 10^-100^	CONTAINS InterPro DOMAIN/s: Methyltransferase FkbM (InterPro:IPR006342); Has 1073 Blast hits to 1073 proteins in 243 species: Archae - 45; Bacteria - 509; Metazoa - 0; Fungi - 4; Plants - 60; Viruses - 4; Other Eukaryotes - 451 (source: NCBI BLink). | chr2:11344003-11345288 REVERSE LENGTH = 319
6070	AT3G28910.1	3 × 10^-84^	ATMYB30, MYB30 | myb domain protein 30 | chr3:10911443-10912856 FORWARD LENGTH = 323
24826	AT3G07800.1	2 × 10^-85^	Thymidine kinase | chr3:2489944-2490935 REVERSE LENGTH = 238

**Table 2:** Gene ontology terms enriched for the 18 gene set validated for analysis using AraNet

Locus_ID	Gene Symbol	GO Plant terms	GO Cellular terms	GO Function terms
AT4G26000	na	shoot development; gynoecium development;	Na	nucleic acid binding;
AT3G07800	na	Na	Na	thymidine kinase activity;
AT1G56580	na	Na	Na	Na
AT5G23850	na	Na	Na	Na
AT5G14450	na	lipid metabolic process;	cellulose and pectin-containing cell wall;	carboxylic ester hydrolase activity;
AT5G19300	na	Na	Na	na
AT2G45640	na	response to salt stress; response to abscisic acid stimulus;	mitochondrion;	protein binding; transcription regulator activity;
AT1G80480	na	Na	plastid chromosome;	na
AT5G59310	LTP4	lipid transport; response to abscisic acid stimulus;	endomembrane system;	lipid binding;
AT5G63090	LOB	organ boundary specification between lateral organs and the meristem;	chloroplast;	na
AT1G69830	na	starch catabolic process;	chloroplast;	alpha-amylase activity;
AT3G28910	MYB30	response to bacterium; hypersensitive response; response to salt stress; response to ethylene stimulus; response to auxin stimulus; response to abscisic acid stimulus; response to gibberellin stimulus; response to salicylic acid stimulus; response to jasmonic acid stimulus; response to cadmium ion;	nucleus;	DNA binding; transcription factor activity;
AT5G63660	na	defense response;	endomembrane system;	na
AT4G25720	na	Na	mitochondrion;	catalytic activity;
AT1G16070	na	regulation of transcription;	Na	transcription factor activity;
AT2G26680	na	Na	endomembrane system;	na
AT1G13260	RAV1	regulation of transcription, DNA-dependent; response to brassinosteroid stimulus; negative regulation of flower development; leaf development; lateral root development;	nucleus;	DNA binding; transcription factor activity;
AT3G58690	na	protein amino acid phosphorylation;	endomembrane system;	kinase activity;

Na = not available.

**Figure 6:**
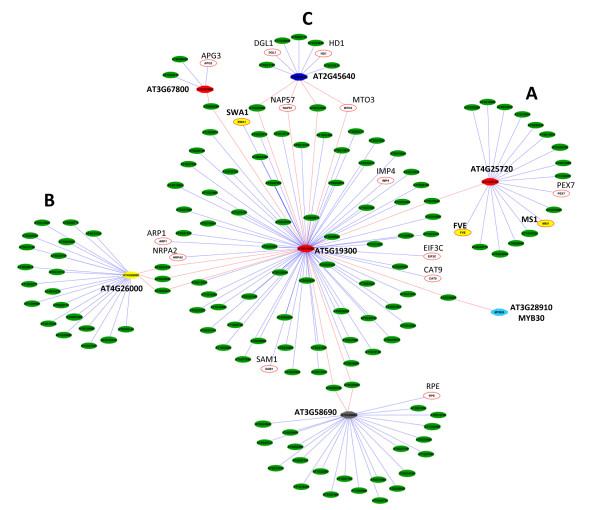
**Prediction of the extended regulatory network of genes that are negatively correlated with BvRAV1-like**. The network was based on analysis of homologous Arabidopsis proteins and constructed using AraNet at http://www.functionalnet.org/aranet Red nodes are unknown, yellow nodes are associated with reproductive growth; grey node is associated with protein modification; dark blue node with histone modification and the light blue node with hormonal signalling. White nodes with red outline represent loci of known genes with GO terms associated with processes including cold regulated biosynthesis (MTO3); amino acid/protein transport (PEX7, CAT9); translation, rRNA processing/biogenesis (EIF3C, IMP4, HD1, NRPA2, APG3, NAP570; ethylene induced biosynthesis (SAM1) embryonic development leading to seed dormancy (ARP1, RPE) and cell wall biogenesis (DGL1). MTO3 = Methionin Over-Accumulator 3; PEX7 = Peroxin 7; CAT9 = Cationic amino Acid Transporter 9; EIF3C = Eukaryotic translation initiation Factor 3 C; HD1 = Histone Deacetylase 1; NRPA2 = DNA binding/DNA-directed RNA polymerase/ribonucleoside binding; APG3 = Albino and Pale Green; SAM1 = S-Adenosylmethionine Synthetase 1; ARP1 = Arabidopsis Ribosomal Protein 1, RPE = Ribulose Phospate 3-Epimerase; DGL1 = dolichyl-diphosphooligosaccharide-protein glycotransferase.

Underlined gene loci are connected in the extended regulatory network illustrated in Figure 6. See also the GO analysis data in Additional file 6

**Table 3:** Plant ontology terms enriched for 20 query genes (excluding *RAV1*) and top 200 new candidates revealed in AraNet.

Rank	ID	Description	p-value	Adjusted p-value	N	m	n	k
1	PO:0007095	LP.08 eight leaves visible	8.88 × 10^-22^	3.28 × 10^-19^	27029	220	12122	168
2	PO:0009052	Pedicel	1.85 × 10^-21^	3.42 × 10^-19 ^	27029	220	13566	178
3	PO:0007098	LP.02 two leaves visible	4.03 × 10^-21^	4.96 × 0^-19 ^	27029	220	12268	168
4	PO:0009006	Shoot	9.21 × 10^-21^	8.50 × 10^-19 ^	27029	220	12752	171
5	PO:0020030	Cotyledon	2.23 × 10^-20^	1.37 × 10^-18 ^	27029	220	12304	167
6	PO:0008019	leaf lamina base	2.46 × 10^-20^	1.37 × 10^-18 ^	27029	220	12580	169
7	PO:0001078	E expanded cotyledon stage	2.78 × 10^-20^	1.37 × 10^-18 ^	27029	220	13839	178
8	PO:0001185	C globular stage	2.99 × 10^-20^	1.37 × 10^-18 ^	27029	220	13704	177
9	PO:0000013	cauline leaf	3.47 × 10-20	1.37 × 10^-18 ^	27029	220	12885	171
10	PO:0004507	D bilateral stage	3.71 × 10^-20^	1.37 × 10^-18 ^	27029	220	13726	177
11	PO:0007115	LP.04 four leaves visible	5.20 × 10^-20 ^	1.74 × 10^-18^	27029	220	13619	176
12	PO:0020038	Petiole	6.00 × 10^-20 ^	1.85 × 10^-18 ^	27029	220	12403	167
13	PO:0001054	4 leaf senescence stage	7.76 × 10^-20^	2.20 × 10^-18 ^	27029	220	12831	170
14	PO:0001081	F mature embryo stage	1.05 × 10^-19 ^	2.76 × 10^-18^	27029	220	13272	173
15	PO:0009010	Seed	1.42 × 10^-19^	3.50 × 10^-18 ^	27029	220	14008	178
16	PO:0020137	leaf apex	2.38 × 10^-19 ^	5.50 × 10^-18 ^	27029	220	12811	169
17	PO:0007103	LP.10 ten leaves visible	3.94 × 10^-19 ^	8.55 × 10^-18 ^	27029	220	12595	167
18	PO:0009009	Embryo	5.61 × 10^-19 ^	1.06 × 10^-17 ^	27029	220	14737	182
19	PO:0007064	LP.12 twelve leaves visible	5.64 × 10^-19 ^	1.06 × 10^-17 ^	27029	220	12106	163
20	PO:0007123	LP.06 six leaves visible	5.72 × 10^-19 ^	1.06 × 10^-17 ^	27029	220	12501	166
21	PO:0009032	Petal	6.26 × 10^-19^	1.10 × 10^-17 ^	27029	220	14601	181
22	PO:0009025	Leaf	1.48 × 10^-18 ^	2.49 × 10^-17 ^	27029	220	14991	183
23	PO:0000230	inflorescence meristem	3.71 × 10^-18 ^	5.96 × 10^-17 ^	27029	220	12965	168
24	PO:0009047	Stem	9.59 × 10^-18 ^	1.48 × 10^-16 ^	27029	220	14033	175
25	PO:0020100	Hypocotyls	2.22 × 10^-17^	3.28 × 10^-16^	27029	220	14126	175
26	PO:0000037	shoot apex	9.61 × 10^-17^	1.36 × 10^-15^	27029	220	14291	175
27	PO:0009029	Stamen	1.23 × 10^-16^	1.68 × 10^-15^	27029	220	13898	172
28	PO:0009031	Sepal	1.89 × 10^-16^	2.49 × 10^-15^	27029	220	15385	182
29	PO:0008034	leaf whorl	2.07 × 10^-16^	2.63 × 10^-15^	27029	220	15696	184
30	PO:0007611	petal differentiation and expansion stage	1.70 × 10^-15^	2.09 × 10^-14^	27029	220	16404	187
31	PO:0009005	Root	1.78 × 10^-15^	2.12 × 10^-14^	27029	220	14917	177
32	PO:0009046	Flower	6.43 × 10^-15^	7.42 × 10^-14^	27029	220	16565	187
33	PO:0007616	4 anthesis	8.20 × 10^-15^	9.17 × 10^-14^	27029	220	16135	184
34	PO:0009030	Carpel	9.14 × 10^-14^	9.92 × 10^-13^	27029	220	13867	166
35	PO:0020091	male gametophyte	2.77 × 10^-10^	2.92 × 10^-09^	27029	220	12609	148
36	PO:0000293	guard cell	1.10 × 10-^06^	1.13 × 10^-05^	27029	220	1815	35
37	PO:0000084	sperm cell	1.35 × 10^-05^	0.000135	27029	220	5341	69
38	PO:0020092	female gametophyte	0.000703	0.006825	27029	220	22	3
39	PO:0007131	seedling growth	0.001218	0.01152	27029	220	862	16

A total of 39 out of 369 terms were enriched by adjusted p value < 0.05

Description of columns: [Rank] [ID] [Description] [p-value (by Hypergeometric test)] [Adjusted p-value (by False discovery rate)] [N = # of total Arabidopsis genes] [m = # of query genes] [n = # of genes for the PO term] [k = # of genes for intersection between m and n]

Network analysis revealed that 10 of the 18 genes in the AraNet database were highly connected within extended regulatory networks, the largest of which included 7 query genes, connected as shown in Figure 6. The unknown gene locus AT5G19300 was the central node, with links to loci broadly involved with translation, rRNA biogenesis and assembly, protein modification, signalling, hormone and cold responses. A gene involved in reproductive organogenesis encoding the WD40 domain protein SLOW WALKER1 (SWA1), which is known to mediate mitotic cell division during female gametogenesis [37] was also directly connected to this central AT5G19300 node. A branch of the network extending to AT4G25720 (Figure 6A) included well established floral transcription factors such as the retinoblastoma-associated protein FVE which regulates flowering time [38]; and the PHD-type transcription factor MALE STERILITY1 (MS1), which regulates pollen and tapetum development [39]. A second extension of the network converged on AT4G26000 (Figure 6B), known to be associated with GO plant terms for gynoecium and shoot development (Table 2 & Additional file 8), while others converged on AT2G45640 largely associated with GO plant terms for cold and hormone regulated responses and including histone modification affecting traits such as juvenility, apical dominance and floral organ development (Table 2 & Additional file 8) and as supported by the presence of HISTONE DEACETYLASE1 (HD1) [40] in this branch of the network (Figure 6C). Of note, histone methylation has also recently been demonstrated to play a role in vernalization induced bolting in sugar beet [41].

### Responses to GA are not highly dynamic in the GA treated apices

**Figure 7:**
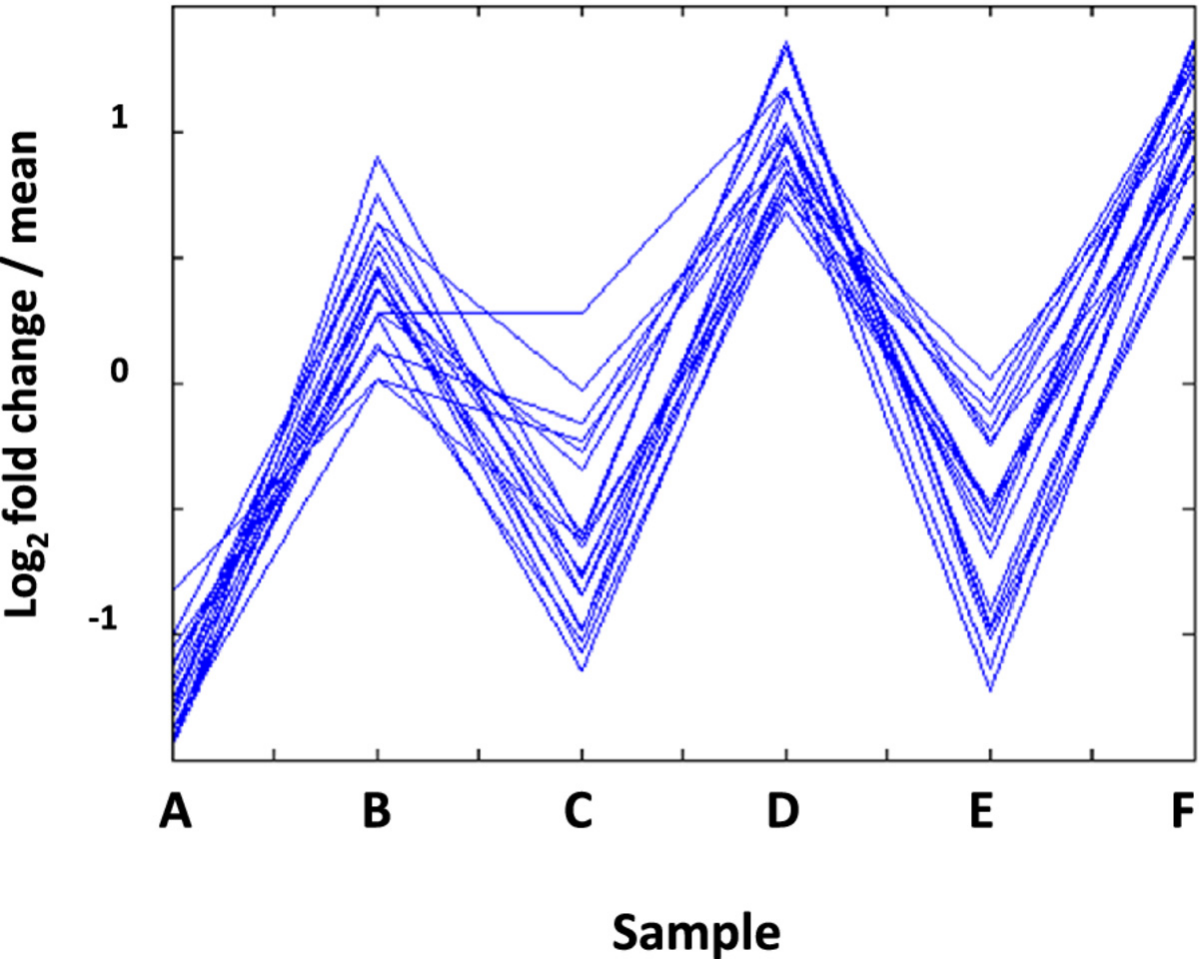
**Expression profiles of genes with robust GA induction under all experimental conditions**. All loci that are consistently up-regulated by GA and displaying similar expression profiles across all 6 samples. A = Sample C600; B = Sample C600/+GA; C = Sample Roberta; D = Sample Roberts/+GA; E = Sample C600/vern; F = Sample C600/vern + GA.

None of our candidate genes revealed highly dynamic differences in response to GA treatment. Therefore, we interrogated our dataset for all those gene loci that displayed similar expression levels across all three non-GA treated samples (C600; C600/vern and Roberta), and at the same time, at least a 2-fold increase in expression following the application of GA. These loci were therefore expected to represent those genes whose expression reflects a generalised response to GA treatment, irrespective of the genetic background. This analysis identified 19 gene loci with robust GA-induction under all three experimental conditions (see Figure 7), thus demonstrating the utility of our DGE dataset for the identification of gene sets of interest without the need to have any prior candidate gene information.

Results of BLASTX searches with these GA-induced genes are given in Table 4, showing that amongst the sugar beet transcripts with significant matches to known genes were included an acetoacetyl-CoA thiolase (Locus 12027), a GA-regulated gene (Locus 30091); and 3 genes encoding efflux-type pumps of the ATP-binding cassette (ABC) transporter (Locus 43376), and multidrug and toxin extrusion (MATE; Locus 54049) families of proteins. Two genes (Locus 39283 and Locus 6420) had weaker homologies with known counterparts but were nevertheless interesting in that protein kinases (e.g. Locus 39283) may have roles in GA-dependent signalling, whilst arabinogalactan proteins (e.g. Locus 6420), are known to have a role in apical cell expansion in the moss *Physcomitrella patens *[42]; in the acceleration of elongation in Arabidopsis root meristems [43] and, one GA-induced protein is known to participate in stem elongation [44]. Just over half (12/19) of the GA-induced genes are unknown, amongst which only 1 (Locus 10708), had a significant BLASTX hit to an unidentified *Vitis vinifera *protein. Interestingly, both the sugar beet and *V. vinifera *genes encode proteins containing a Mediator complex subunit 27 (Med27) super family conserved domain. Med27 proteins are well recognised co-factors that mediate the association of transcription factors with the basal transcriptional machinery to modulate the activity of RNA polymerase II [45,46].

**Table 4:** Table to show the list of loci up-regulated by GA application, together with predicted annotations based on BLASTX hits in public databases

Locus ID	Transcript Length (bp)	BLASTX best hits DB Accessions	p-value	Annotation
12027	1800	ABV08820.1	0	acetoacetyl-coenzyme A thiolase [*Salviamiltiorrhiza*]
		ABC74567.1	0	acetoacetyl-CoA thiolase [*Picrorhiza kurrooa*]
		NP_568694.2	0	acetyl-CoA acetyltransferase, cytosolic 1 [*Arabidopsis thaliana*]
		AAU95618.1	0	cytosolic acetoacetyl-coenzyme A thiolase [*Nicotiana tabacum*]
30091	1389	AAW83819.1	6 × 10^-49^	GASA2-like protein [*Pelargonium zonale*]
		CBI30071.3	6 × 10^-48^	unnamed protein product [*Vitis vinifera*]
		XP_002276458.1	8 × 10^-48^	PREDICTED: hypothetical protein [*Vitis vinifera*]
		ABQ42002.1	5 × 10^-46^	gibberellin induced protein [*Sonneratia caseolaris*]
		ABD33300.1	9 × 10^-45^	Gibberellin regulated protein [*Medicago truncatula*]
		AEC10958.1	1 × 10^-43^	gibberellin induced protein [*Camellia sinensis*]
43376	829	AAD10836.1	3 × 10^-50^	P-glycoprotein [Solanum tuberosum]
		XP_002323485.1	6 × 10^-48^	multidrug/pheromone exporter, MDR family, ABC transporter family [*Populus trichocarpa*]
		ABB97035.1	4 × 10^-47^	ABC transporter-like protein [*Brassica rapa*]
54049	574	ACU23396.1	2 × 10^-56^	unknown [Glycine max]
		XP_002532163.1	1 × 10^-48^	DNA-damage-inducible protein f, putative [*Ricinus communis*]
		NP_001164052.1	2 × 10^-44^	MATE family protein [*Zea mays*]
		XP_002891631.1	1 × 10^-43^	mate efflux family protein [*Arabidopsis lyrata *subsp. *lyrata]*
		ADK70243.1	2 × 10^-43^	aluminum activated citrate transporter 1-5 [*Secale cereale*]
		BAD87624.1	7 × 10^-39^	MATE efflux family protein-like [*Oryza sativa *Japonica Group]
73039	873	NP_001151695.1	2 × 10^-49^	gamma-interferon-inducible lysosomal thiol reductase [*Zea mays*]
		NP_563779.1	3 × 10^-48^	GILT domain-containing protein [Arabidopsis thaliana]
6420	936	NP_974828.1	6 × 10^-08^	arabinogalactan protein 41 [*Arabidopsis thaliana*]
39283	597	XP_002532467.1	5 × 10^-10^	Protein kinase APK1B, chloroplast precursor, putative [*Ricinus communis*]
		XP_002888501.1	4 × 10^-06^	kinase family protein [*Arabidopsis lyrata *subsp. *lyrata*]
10708	1591	XP_002274655.1	2 × 10^-156^	PREDICTED: hypothetical protein [*Vitis vinifera*]
27762	238			Unknown *Beta vulgaris *subsp. *vulgaris*
29113	532			Unknown *Beta vulgaris *subsp. *vulgaris*
32928	100			Unknown *Beta vulgaris *subsp. *vulgaris*
54739	168			Unknown *Beta vulgaris *subsp. *vulgaris*
79469	143			Unknown *Beta vulgaris *subsp. *vulgaris*
88173	286			Unknown *Beta vulgaris *subsp. *vulgaris*
94815	102			Unknown *Beta vulgaris *subsp. *vulgaris*
127929	100			Unknown *Beta vulgaris *subsp. *vulgaris*
136937	100			Unknown *Beta vulgaris *subsp. *vulgaris*
151222	100			Unknown *Beta vulgaris *subsp. *vulgaris*
174489	100			Unknown *Beta vulgaris *subsp. *vulgaris*

### Data access

All raw sequencing data have been submitted to the European sequencing archive at the European Bioinformatics Institute under accession number EBI: ERP000947. In addition, a number of supplementary data files are attached to this manuscript including the transcriptome assembly used for the analysis presented above (Additional file 2), the digital gene expression profiling table (Additional file 4) and files containing the data plotted in Figures 4 and 5 (Additional files 5 and 7). To enhance utility of the transcriptome data generated within this paper, we have generated two Additional files. The first one (Additional file 1) contains the results of further processing of our transcriptome assembly and was generated using the Minimus assembly tool [22]. As such, it represents a consolidation of the transcripts into a smaller number of overall contigs and may therefore be particularly useful for the identification of near full length transcripts. As with all RNA-Seq assemblies, careful case-by-case evaluation of transcripts will be advisable given the possibility that highly related genes may have been inadvertently merged [47], a particular concern given the substantial expansion of gene families commonly seen in crop plants [48,49]. The final file (Additional file 3) contains a complete mapping of our transcripts to the Arabidopsis proteome, as a searchable tab delimited file. This file will be particularly useful for the sugar beet research community as it provides streamlined access to primary sugar beet mRNA sequences that correspond to a given Arabidopsis protein.

## Discussion

The application of next generation sequencing technology to RNA/cDNA sequencing (RNA-Seq) provides a rapid and cost-effective way to obtain large amounts of transcriptome data for any type of biological sample for which reasonable amounts of RNA can be extracted. One important consequence of these developments is that transcriptome-scale investigations, which were hitherto limited to a small range of model organisms, can now be employed much more widely. This includes the study of agronomically important crop plants. Here, we have used RNA-Seq to generate the first shoot apex transcriptome for sugar beet, and to study the transcriptional response to vernalization and GA treatment at the transcriptome-scale.

Within the field of RNA-Seq, the development of new sequencing platforms as well as bioinformatic tools for reference assembly and mapping is an area of active investigation with constant development of new sequencing technology and data processing algorithms. Consequently there is as yet no definitive consensus on a single best approach. The application of short reads is well proven in other non-model systems [18-20] and we expect may become the method of choice, as it currently provides the best value for money. Nevertheless, it is likely that future algorithm development will improve upon current sequence assembly tools for the assembly of transcriptome data from 100 bp reads. It may indeed be that one day; a gold standard will be established. Since all our data are publicly accessible, we hope that they may be helpful in contributing towards that goal, and in any case will be available for re-analysis by the wider scientific community.

### A sugar beet reference transcriptome generated by RNA-seq

As for many other agronomically important crop plants, the development of genomics resources for sugar beet has lagged far behind model organisms, with no public reference genome or expression array platforms reported to date. However, the compilation of a sugar beet gene index from a variety of EST collections such as those generated by [50-53], has provided a valuable resource. It was therefore necessary to benchmark the RNA-Seq-derived transcriptome generated here against this existing sugar beet gene index. This analysis revealed several important points:

i) Approximately 75% of the 17,186 unigene ESTs in the sugar beet gene index had a counterpart in our RNA-Seq transcriptome. This demonstrated that a large proportion of genes previously defined using traditional sequencing technologies were recovered in our RNA-Seq based study.

ii) 25% of previously known sugar beet unigene ESTs were not recovered which may be indicative of our tissue type and/or conditions to which the plants were subjected. Further, RNA-Seq based studies of additional sugar beet tissues are likely to increase the proportion of ESTs recovered by RNA-Seq.

iii) We identified 7,468 large transcript loci with no counterparts in the sugar beet gene index collection. Since these loci contain transcripts assembled from at least 6 independent 100 bp reads, extending over a minimum of 500 bp of mRNA sequence, the majority of these loci can be expected to represent *bona fide *protein-encoding mRNA. Notwithstanding the possibility that some of these loci could be the result of mis-assemblies, our data may now have expanded the total known gene count of sugar beet by up to 40%. Such an enlarged gene count will not only facilitate future efforts to assemble a sugar beet reference at the genome level, but also represents an immediately exploitable resource for gene discovery by both basic scientists and the sugar beet breeding community. The original data sets may be accessed in the sequence read archive (SRA) database at EBI under study accession number EBI: ERP000947.

As a general resource to the wider sugar beet community, the reference transcriptome described here is limited by having been generated not from the whole plant but from a single tissue type and under specified treatments and conditions. As a resource, it is therefore most suited to the analysis of reproductive developmental transitions in the shoot apex and hence for applications in genetic crop improvement for bolting and flowering time control. It is widely recognised that Illumina HiSeq technology can generate a significant amount of reads that are difficult to incorporate in the final assembly with currently available bioinformatics tools (~23%-29% with our samples). This suggests that our assembly could improve further as new tools come on line.

### Digital gene expression profiling is a powerful tool for new gene discovery & functional annotation

Reverse genetic-based approaches for the identification of gene candidates associated with key agronomic traits in crop plants are useful and informative. However, they may be encumbered by the need for transgenic analyses in crop plants, some of which may be recalcitrant to transformation. Expression assays of target genes may also be limited by the ability to design suitable qRT-PCR primers. Here, we have demonstrated the utility of next generation sequencing-based DGE profiling, not only as a tool for screening known gene candidates, but also for selectively identifying genes that are directly regulated by specified cues. For example, a MADS box transcription factor was revealed to be strongly up-regulated by vernalization. This was unexpected, since in dicotyledonous plants, MADS box transcription factors examined so far are repressors of floral induction that are known to be down-regulated by vernalization, as exemplified by *FLC *[14,54]. Nevertheless, we cannot discount the possibility that MADS box proteins may have a different role in sugar beet, indeed, in the shoot apex transcriptome, we found that the sugar beet *FLC *homologue, (*BvFL1 *-[55]) is not down-regulated by vernalization (data not shown). Further, the discovery that transcripts which encode an AP2/B3 BvRAV1-like protein are strongly up-regulated by vernalization, now establish a role for a member of this protein family in vernalization responses for the first time. In the vernalized sugar beet apex transcriptome, we identified at least 14 other transcripts encoding AP2/B3 domain proteins (data not shown), none of which were similarly up-regulated by vernalization. The distinctive behaviour of BvRAV1-like therefore suggests a different role in vernalization which may extend beyond the generalized role in cold stress responses as for *AtRAV1 *[34,35,56]. Transcriptome-scale analysis also enabled inference of putative gene functions of co-regulated transcripts, providing further support of a role for BvRAV1 in vernalization-induced reproductive growth processes in sugar beet. Thus, although the type of network analysis based on inference from co-regulated genes as described for BvRAV1 may be regarded as speculative, it nevertheless provides a rapid and comprehensive approach to candidate gene discovery. Hence, our discovery of BvRAV1 as a likely regulator of vernalization provides an attractive new hypothesis, which certainly will require further experimental validation, but nevertheless already adds an interesting new dimension to the identification of novel breeding targets for the sugar beet crop.

Another powerful application of DGE data, in cases such as that described here where expression array platforms and reference genomes are not available, is the ability to selectively identify, at the transcriptome-scale, those genes that behave in a specified manner under given conditions. Thus, we discovered 19 sugar beet genes that are directly up-regulated at least 2-fold by GA treatment of shoot apices. Although our analysis was conducted in a manner expected to reveal genes whose expression patterns would represent a general response to GA treatment, we cannot exclude the possibility that a different set of genes might be revealed in experiments with alternative genotypes. Nevertheless, for the genes discovered here, amongst the 8 genes that we putatively annotated using BLASTX, four appear to be consistent with the expected physiological effect of applied GA. For example, the added GA is well in excess of normal physiological levels hence, we might speculate that an immediate response of the plant may be to actively remove the excess GA. Here, we found that two of the GA-induced genes are predicted to have efflux pump associated functions, as represented by the ABC transporter and MATE family protein genes [57,58]. The unknown Med27 domain protein gene is also plausible since GA participates in the hormonal orchestration of transcriptional regulation. It is therefore encouraging to find that one of the GA-responses in the sugar beet shoot apex appears to involve the up-regulation of a protein with strong similarity to a well characterised family of co-transcription factors. Detailed analysis of the remaining, as yet unknown genes will, in future, reveal new insight into the molecular basis of GA-dependent bolting and flowering mechanisms in sugar beet.

## Conclusions

Here, we have shown that next-generation sequencing technology permits quantitative analysis of gene expression in sugar beet, at the level of the whole transcriptome, without the need for a reference genome or established array platforms. Comprehensive bioinformatic analysis identified transcriptional programmes associated with different genotype as well as biological treatments; thus, providing a wealth of new opportunities for both basic scientists and sugar beet breeders. In addition to applications for addressing basic biological questions, as described in this manuscript, next generation reference transcriptomes will also be very useful for the assembly of the anticipated crop reference genomes.

We believe that our approach is widely applicable to other crop species and will be ideally suited to the study of key agronomic traits which, in future, will certainly be driven by the food security agenda. The demonstrated ability to generate robust reference transcriptome assemblies and digital gene expression profiling, coupled with the decreasing costs of next generation sequencing technologies, will make this method ever more accessible. Indeed, it may become the routine workhorse for a systems-based approach in those crops where publicly available genomic resources are limited. Specifically for the sugar beet crop, this manuscript lays the foundation for future detailed analysis of bolting mechanisms at the molecular level.

## Methods

### Plants

C600 MB1 sibA plants were raised from seeds generated in-house from a sibling cross whose original biennial parent was propagated by single seed decent from a line that was segregating for vernalization requirement. Genotype at the bolting locus (*B*-gene) was confirmed using a PCR marker as previously described [8]. The original C600 lines were a kind gift from Bob Lewellen, (Plant Geneticist, USDA ARS, Salinas, California - now retired), and are closely related to the previously described C600 line, PI 520748 [59].

Roberta is our lab standard commercial sugar beet cultivar, which in common with most commercial genotypes, is a combination of 3 genetic backgrounds and has an obligate requirement for vernalization. Although this is a legacy cultivar it is representative of current European varieties and is kindly maintained and provided to us by Gunter Diener at KWS SAAT AG, Einbeck, Germany.

### Plant growth and cultivation

Plants were grown and vernalized as previously described [6] except that photoperiod was set to 8 h light and they were vernalized for 21 weeks. Non-vernalized plants were chronologically younger than vernalized plants but considered to be at the same developmental stage as determined by the number of fully expanded leaves at apex harvest. During the experiment, plants were kept in a controlled environment room at 22°C, under short day (SD) conditions (8 h light) in light intensities of ~285 μmol m^-2 ^s^-1 ^of photosynthetically active radiation (PAR).

### GA_4 _application, apex harvest and RNA extraction

GA treatment started at 5 weeks post vernalization and was continued for two weeks immediately prior to the harvest date. In this case, GA_4 _was applied to plant shoot apices (10 μL of 200 μM stock) on alternate days and always at 7.5-8 h after lights came on, being the time since the environmental cue (ZEITGEBER (ZT); in this case light) that sets the circadian clock is expressed, entraining biological and hence expression rhythms in the plants. Figure 1B i) - iii) shows the appearance of plants immediately prior to harvest, which was carried out consistently at 4 hours after the lights came on (ZT4), by harvesting plant shoot apices (Figure 1B iii)) into 50-mL Falcon tubes, and keeping them cool on ice until the shoot apices were dissected out as indicated (Figure 1B iv), while taking care to remove all leaf and vascular tissues as visualised under the stereo dissection microscope. Dissected apices were transferred to RNALater and stored at 4°C until total RNA was extracted using the Qiagen Plant RNAeasy Kit. Apex tissues were disrupted using ceramic beads in the Bertin Technologies Precellys 24 Lysis & Homoginization machine (supplied by Stretton Scientific Ltd, UK), and the Qiagen RLT buffer was selected for RNA extraction. The RNA was DNAse treated with Ambion DNA-free™ DNase Treatment and quantified using the NanoDrop 2000/2000c (Thermo Fisher) machine.

### mRNA sequencing and cDNA *de novo *assembly

To create a reference sequence, aliquots of total RNA (~5 μg) from each sample (Figure 1A) were pooled and used to create a custom normalised cDNA library which was sequenced by Illumina HiSeq2000 (100 bp single read module) and processed for assembly into contigs and loci using the software tools Velvet/Oases (v1.04 and v0.1.21 respectively). Sequencing and assembly were provided as a custom service by Eurofins MWG GmbH, Ebersberg, Germany. A schematic of the process is shown in Figure 2A. To evaluate the selected assembly tools further, the reads were re-assembled in-house, using Minimus [22].

### Digital gene expression profiling and mapping to reference

cDNA libraries generated from sub-samples of each test sample were sequenced (Illumina HiSeq2000, 50 bp single read module). Reads for each sequence tag were counted and mapped back to the reference using the software tool Bowtie [28]. cDNA library construction, sequencing DGE profiling and mapping back to the reference transcriptome as above were carried out as a custom service by Eurofins MWG GmbH. The outsourcing of these tasks, to highly experienced and reputable service providers, and in particular library construction, is recommended to reduce the risk of potential technical problems which may introduce bias in the final data sets.

### Validation of the reference assembly

Reference transcripts assembled from the 6.6 Gb of total reads from the pooled cDNA samples were validated by direct comparison with EST sequences in the sugar beet gene index data base at http://compbio.dfci.harvard.edu/cgi-bin/tgi/gimain.pl?gudb=beet using BLAST, set to a stringency of 100 bp overlap and ≥ 98% sequence identity. Further validation was obtained by comparison with peptide sequences in the Arabidopsis TAIR database (version TAIR10), using BLAST and an E-value cut off of 1 × 10^-10^. These analyses were carried out in-house.

### Analysis of expression profiles in experimental samples

A matrix was generated with tag counts for each locus in each sample, and normalised to counts per 10 million reads [(reads/total reads per sample) × 10 million]. Only those loci with tag counts of 10 or above in at least one experimental sample were retained for further analysis. All correlation analyses, hierarchical clustering and principal component analysis were performed using Matlab http://www.mathworks.com. This was carried out in-house.

## Competing interests

The authors declare that they have no competing interests.

## Authors' contributions

ESM-G conceived and designed the study; carried out experiments and wrote the manuscript. AJ carried out the bioinformatics and statistical analyses and contributed to writing the manuscript. HFH carried out experiments and contributed to writing the manuscript. PH contributed to the conception and design of the study and to writing the manuscript. BG directed and contributed to the bioinformatics and statistical analyses and to writing the manuscript. All authors read and approved the final manuscript

## Supplementary Material

Additional file 1 **Minimus second pass assembly output data**. Click here for file

Additional file 2 **Sugar beet shoot apex transcriptome assembly- Illumina 100 bp reads**. Generated from C600 and Roberta Genotypes with and without vernalisation and/or applied GA. Click here for file

Additional file 3 **Annotation of the sugar beet shoot apex reference transcriptome**. All loci were mapped to the Arabidopsis proteome (version TAIR10 database) by BLASTX comparison with a cut off E-value of 1 × 10^-10^. Click here for file

Additional file 4 **DGE tag count matrix for all loci**. Normalized DGE profile tag counts for all loci with a count of 10 in at least one of the test samples. Click here for file

Additional file 5 **Differentially expressed loci for C600 *vs *Roberta genotypes**. Click here for file

Additional file 6 **Supplementary Figure 1**. BvRAV1-like/AtRAV1 alignment and BvRAV1-like conserved protein domains. Click here for file

Additional file 7 **List of gene loci that are positively and negatively correlated with *BvRAV1-like *at a probability of p > 0.95**. Click here for file

Additional file 8 **Gene Ontology and InterPro Domain enrichment analyses**. For genes that are negatively correlated with BvRAV1 together with their associated candidates as revealed by network analysis using AraNet. Click here for file
