# Supplementary material for: A new RNASeq-based reference transcriptome for sugar beet and its application in transcriptome-scale analysis of vernalization and gibberellin responses
Source: BMC Genomics. 2012 Mar 19;13:99. doi: 10.1186/1471-2164-13-99 (PMC3340327; doi:10.1186/1471-2164-13-99)
Supplement: Additional file 6 — Supplementary Figure 1. BvRAV1-like/AtRAV1 alignment and BvRAV1-like conserved protein domains. [file 1471-2164-13-99-S6.PPT]

## Slide 1
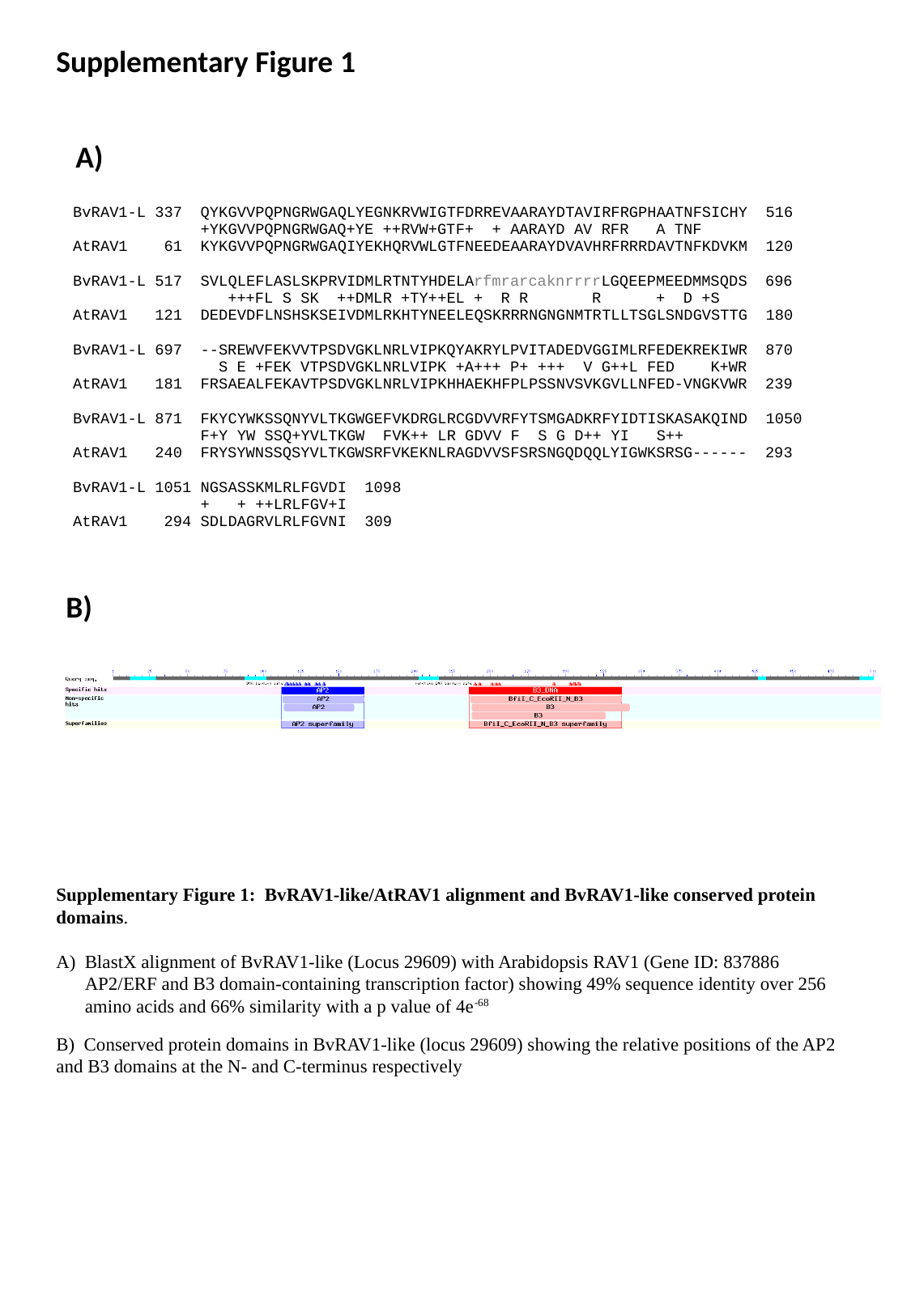

Supplementary Figure 1
A)
B)
Supplementary Figure 1: BvRAV1-like/AtRAV1 alignment and BvRAV1-like conserved protein domains.
BlastX alignment of BvRAV1-like (Locus 29609) with Arabidopsis RAV1 (Gene ID: 837886 AP2/ERF and B3 domain-containing transcription factor) showing 49% sequence identity over 256 amino acids and 66% similarity with a p value of 4e-68
B) Conserved protein domains in BvRAV1-like (locus 29609) showing the relative positions of the AP2 and B3 domains at the N- and C-terminus respectively
